# Does Grammatical Structure Accelerate Number Word Learning? Evidence from Learners of Dual and Non-Dual Dialects of Slovenian

**DOI:** 10.1371/journal.pone.0159208

**Published:** 2016-08-03

**Authors:** Franc Marušič, Rok Žaucer, Vesna Plesničar, Tina Razboršek, Jessica Sullivan, David Barner

**Affiliations:** 1 Center for Cognitive Science of Language, University of Nova Gorica, Nova Gorica, Slovenia; 2 Department of Psychology, Skidmore College, Saratoga Springs, New York, United States of America; 3 Department of Psychology, University of California San Diego, La Jolla, California, United States of America; 4 Department of Linguistics, University of California San Diego, La Jolla, California, United States of America; University of Western Ontario, CANADA

## Abstract

How does linguistic structure affect children’s acquisition of early number word meanings? Previous studies have tested this question by comparing how children learning languages with different grammatical representations of number learn the meanings of labels for small numbers, like 1, 2, and 3. For example, children who acquire a language with singular-plural marking, like English, are faster to learn the word for 1 than children learning a language that lacks the singular-plural distinction, perhaps because the word for 1 is always used in singular contexts, highlighting its meaning. These studies are problematic, however, because reported differences in number word learning may be due to unmeasured cross-cultural differences rather than specific linguistic differences. To address this problem, we investigated number word learning in four groups of children from a single culture who spoke different dialects of the same language that differed chiefly with respect to how they grammatically mark number. We found that learning a dialect which features “dual” morphology (marking of pairs) accelerated children’s acquisition of the number word *two* relative to learning a “non-dual” dialect of the same language.

## Introduction

Humans have a unique ability to create precise symbolic representations of natural number, and to use these symbols in the service of mathematics. This ability emerges early in life, when children begin learning verbal labels like *one*, *two*, *three*, *four*, *five*, etc. Members of groups that lack such words, like the Pirahã and Mundurucu, are unable to perform precise numerical computations for large numbers [1]; [2]; [3]; [4]. However, besides the observation that verbal labels appear to be important to acquiring mathematical knowledge, very little is known about the precise causal role that language plays in the development of natural number concepts, or how the particular language that a child learns affects this ability. On some views, linguistic labels drive the construction of new conceptual resources, including concepts like “one”, “two”, and “three” [5]; [6]. Alternatively, words may label pre-existing concepts, such as those that support the acquisition of other grammatical expressions of number, like quantifiers and singular-plural morphology [7]; [8]. Here we examined whether number words share content with representations of grammatical number by asking how these forms are related in development.

In the US, middle class English-speaking children typically begin learning number words at around age 2 by memorizing a partial count list–e.g., *one*, *two*, *three*, *four*, *five*, etc.–which is initially recited as a blind routine, without specific meanings for each number word [9]. Next, children learn an exact meaning for the word *one* (“1-knowers”). When asked for *one fish*, for example, they give 1 fish, but do not give 1 fish when asked for *two* or *three*. Some 6 to 9 months later, they learn an exact meaning for *two* (“2-knowers”). They then learn *three* several months later (“3-knowers”), and then in some cases *four* (“4-knowers”), before a broader shift occurs and children realize that they can use the counting routine to label sets of any size. When asked to give, e.g., *six* objects, these children count the objects, often pointing at them as they do so, and give the requested amount. At this point, they are generally called “Cardinal Principle knowers” or CP-knowers (see [6]; [10]; [11]; for discussion see [12]).

Critical to the current study, just as early number word meanings are limited to a set size of 3 or 4 in absence of counting, a similar limit is found in the grammatical marking of number cross-linguistically. Many languages, like English, feature morphological marking of singular and plural number–e.g., *one box; three box(es)*–such that the singular form is generally used when referring to singleton sets, while the plural is generally used to label pluralities (see [13], for review, and exceptions). Other languages, like Saudi Arabic and Slovenian, make an additional contrast and mark singular, dual, and plural ([14]; [15]; for details see SI). In such languages, the dual form is typically used to describe sets of 2. For example, in Slovenian, the numeral *two* is obligatorily accompanied by dual morphology that is marked throughout the sentence. As shown in example (1), below, the dual is marked on nouns (gumba / button), adjectives (rdeča / red), verbs (ležita / lie), and even on the numeral itself (dva / two). Historically dual languages are very common, and include Sanskrit, Ancient Greek, Biblical Hebrew, Proto-Germanic, Old Irish, and others [14]. Some languages even have a pronominally marked trial form, which is used to describe quantities of three. However, no attested language marks quantities of four or more (see [14]; [16]). Thus, as in the case of number word learning, the morphological paradigms of the world’s languages appear to exhibit a set size limit, and can represent precise quantities up to three, but probably not more.

1.        Slovenian

Dva        rdeča        gumba        ležita        na mizi.

Two_DUAL_        red_DUAL_        button_DUAL_        lie_DUAL_        on table.

‘Two red buttons are lying on the table.’

By some accounts (e.g., [6]; [8]; [17]; [18]), the fact that number morphology and children’s early number word meanings share a set-size limit of three or four provides evidence for shared conceptual representations, perhaps constrained by the limits of visual working memory, which also can represent up to 3 or 4 objects simultaneously. For example, studies by Feigenson and colleagues suggest that infants can keep track of up to 3 or 4 objects in parallel (e.g. [19]), analogous to the adult ability to track 3 or 4 visual objects at a time (e.g. [20]). Also, extensive evidence suggests that humans, like many other species, are able to encode and compare the magnitude of sets approximately, such that noise in representations increases as a function of magnitude according to Weber’s law, permitting relatively precise discrimination of small sets [18]; [21]; [22]; [23]. Such findings support the idea that the concepts encoded by early number words are not domain-specific and unique to mathematics. Instead, the concepts encoded by number words like *one*, *two*, and *three* may be more general and independent of mathematics, and perhaps older than natural language itself.

Additional support for this idea comes from studies which show that grammatical contrasts like that between singular and plural accelerate children’s learning of early number word meanings–suggesting that the same conceptual content is encoded by each system. As already noted, in English and many other singular-plural languages, the word for 1 is typically used with singular agreement (*one cup*), whereas larger number words are used with the plural (*two cups*, *three cups*). Previous studies [5] indicate that children learning English may use this distinction to accelerate their acquisition of the word *one*, by inferring that *one box* refers to a single box (because it is used with singular agreement), whereas *two* and higher numbers refer to sets of more than one (because they occur with plural agreement). In support of this, English-speaking children are significantly faster to learn the word for 1 compared to children learning Mandarin Chinese ([7]; [24]), and Japanese ([25]; [26]), both of which lack obligatory singular-plural marking. Also, surprisingly, children learning Slovenian, which has grammatical marking of the dual, learn the word for 2 many months earlier than children learning any other language, including English, Russian, Japanese, and Mandarin, despite receiving little exposure to counting [7]. This advantage appears to be specifically due to the fact that Slovenian has dual morphology. First, Slovenian children are faster to learn words for 1 and 2, but not the meanings of words for higher numbers, like 3 and 4. This results in a surprisingly high frequency of 2-knowers in Slovenian–about 50% of all children at 2, 3, and 4 years of age (compared to 11%, 20%, and 8% at these ages in English-speaking children). The acquisition of larger numbers is instead predicted by counting ability, which is much stronger in English-speaking children. Second, learning the meaning of the word for 2 in Slovenian is strongly correlated with comprehension of dual morphology: Only children who have learned the dual are faster to learn the word for 2. Finally, qualitatively similar results are also reported for Saudi Arabic, which also has dual marking, but otherwise is unrelated to Slovenian [7]. In their study, almost half of all 3- and 4-year-old children learning Saudi Arabic were 2-knowers.

These past studies are consistent with the hypothesis that informative number morphology accelerates number word learning, and consequently that the conceptual content encoded by children’s early number words may not be uniquely mathematical, but may also arise spontaneously in natural language. Nevertheless, support for this conclusion remains preliminary. The primary problem is that cross-linguistic differences in number word learning, though correlated with differences in grammatical structure, may also be due to other linguistic or cultural differences not measured in these studies. For example, most past studies did not measure individual subjects’ exposure to number words across different cultures, or whether subjects differed in their familiarity with counting routines (cf. [7]). Instead, most studies have used small corpus datasets or broad cultural differences in educational practices to make inferences about children’s likely training histories. More generally, although these initial studies provide suggestive correlational data, any number of unmeasured linguistic and cultural differences might also contribute to reported cross-cultural differences.

In the present study, we provided a stronger test of the hypothesis that informative grammatical structure speeds number word learning. Specifically, we exploited a remarkable typological feature of the Slovenian language: Whereas in Ljubljana, the capital of Slovenia, children learn a dialect of the language that robustly marks the dual, in some other regions children learn dialects that lack dual marking, despite being otherwise similar (see SI for details). In the present study, we sought to replicate the finding that learners of a dual language acquire the meanings of words for 1 and 2 faster than children learning a language that lacks a dual form, by testing a new group of children from Slovenska Bistrica, Slovenia, where the dual is spoken. In addition, we tested children in two regions of Slovenia in which dual morphology is not generally used: Metlika and Nova Gorica. These three new datasets allowed us to compare children from the same country, in the same educational system, and who spoke the same language, but who differed chiefly with respect to their exposure to a dialect with or without dual morphology. We reasoned that if number morphology, rather than other cross-cultural differences, explains cross-linguistic differences in number word learning, then Slovenian children who learn the dual should be faster to learn the meanings of words for 1 and 2, compared to Slovenian children who do not learn the dual.

## Method

### Participants

**Fig 1:**
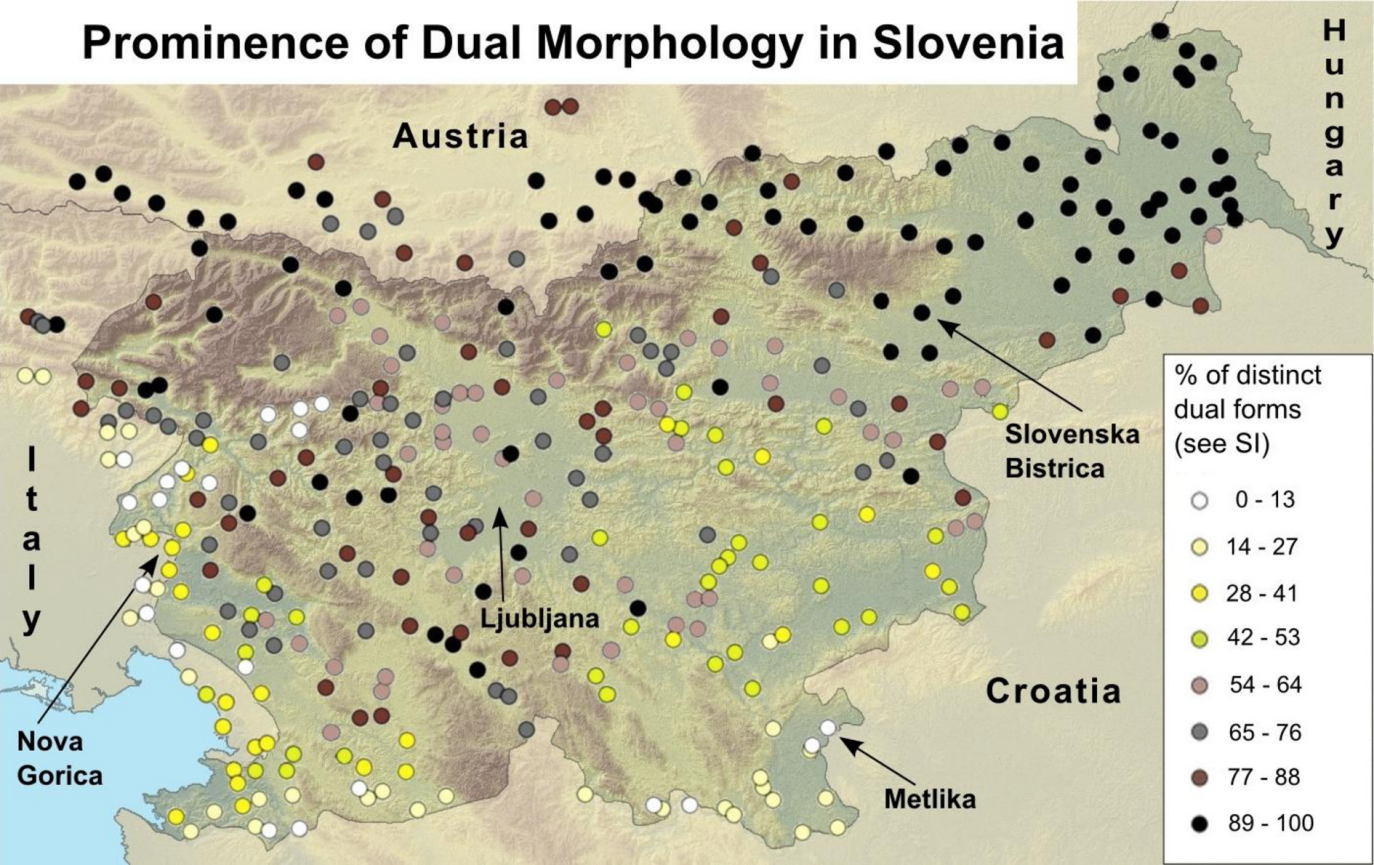
Map of dual variability across Slovenian dialects in Slovenia and neighboring areas from 0–13% penetration of dual usage (white dots) to 89–100% (black dots). See SI for details and source.

Participants were 2-, 3-, and 4-year-old children from three regions of Slovenia: Slovenska Bistrica (*n* = 83), where the dual marker is habitually used, Metlika (*n* = 77) and Nova Gorica (*n* = 81), where dialects of Slovenian without the dual marker are spoken. Sample sizes were chosen in order to match those of previous datasets collected in the US and Slovenia [7]. The Slovenska Bistrica, Metlika, and Nova Gorica samples were selected on the basis of a previous typological study of dual usage in Slovenia [27]. We have re-plotted these data in Fig 1.

Also, we selected these groups from regions with diverse socio-economic profiles. As shown in Table 1, average household incomes in Slovenska Bistrica (dual) and Metlika (non-dual) are lower than the Slovenian national average household income, while those in Nova Gorica (non-dual) and Ljubljana (dual) are above the Slovenian national average, though the average household incomes of Nova Gorica and Slovenska Bistrica are relatively close.

**Table 1:** Average monthly income (in Euros) of residents in Ljubljana, Nova Gorica, Slovenska Bistrica, and Metlika (from the Statistical Office of the Republic of Slovenia (pxweb.stat.si)).

	2008	2009	2010	2011	2012	2013
Ljubljana	1042,35	1081,27	1106,95	1125,64	1124,97	1125,61
Nova Gorica	971,55	994,66	1027,89	1032,97	1022,04	1016,28
Slovenian average	899,80	930,00	966,62	987,39	991,44	997,01
Slovenska Bistrica	846,49	856,83	916,48	930,33	937,91	962,09
Metlika	721,15	749,19	777,11	819,89	812,78	828,38

In addition to these new datasets, we also made use of previously reported data from children learning Central Slovenian in Ljubljana (*n* = 71), where the dual is marked, and from children learning English in San Diego, CA, USA (*n* = 79), where the dual is not marked (both datasets reported in [7]).

All children gave verbal assent, and caregivers gave signed consent. Recruitment and experimental procedures were approved by the UCSD Human Research Protection Program and by the Ethical Committee of the Philosophical Faculty of the University of Ljubljana. Experimenters included speakers of each local dialect.

### Tasks

All Slovenian children completed three tasks: Give-Morphology (Give-M), Give-a-Number (Give-N), and Highest Count, in this order. English-speaking children received the Give-N and Highest Count tasks, in order. For reasons we explain below, children in Ljubljana and Nova Gorica also completed an additional task measuring knowledge of dual morphology–Wynn’s “What’s on this card” task (reported in [7]). Children in Ljubljana also received one additional task that tested the pragmatics of singular and dual forms, which are reported in a separate study. Testing took approximately 15 to 30 minutes depending on which tasks the child received.

### Give-M

All Slovenian children (from Slovenska Bistrica, Nova Gorica, Metlika, and Ljubljana) were tested on their comprehension of dual morphology using the Give-Morphology (Give-M) task. In this task the experimenter presented children with a set of 10 buttons, and asked them to place a quantity into a box, using the singular (*gumb*), dual (*gumba*) or plural form (*gumbe*) of the word for button, without a numeral: “Now we’re going to play a game. There are buttons and there is a box. I want you to put buttons in the box. Listen carefully. Can you put X in the box (*Zdaj se bova igrala/igrali novo igrico*. *To so gumbi in tu je škatlica*. *Rad/rada bi*, *da gumbe prestaviš v škatlico*. *Pozorno poslušaj*. *Ali lahko prestaviš X v škatlico*)?” The experimenter then repeated: “Put X in the box and tell me when you finish (*Prestavi X v škatlico in povej*, *ko boš zaključil/a*).” Whether they gave the correct response or not, all children were told: “Very good (*Zelo dobro*)!” at the end of each trial.

Children received each trial type (singular, dual, plural) four times in fixed, pseudo-random order. Responses were coded as “correct” if children gave 1 object for singular requests, 2 for dual requests, and at least 3 for plural requests. Six children from the Ljubljana sample did not complete this task.

### Give-N

We used the Give-a-Number (Give-N) task to identify children’s “knower level”–i.e., the highest number word for which they had an adult-like meaning [11]. Children were presented 10 buttons, and were asked, “Can you put *N* in the box (*Ali lahko prestaviš* N *v škatlico*)?” where *N* was 1, 2, 3, 4, 5, 8, or 10. This question was phrased such that morphological cues (from singular, dual, or plural forms) were absent and thus could not influence performance. Once the child had placed objects into the box, the experimenter asked, “Is that *N* (*Je to* N)?” If the child said “no” or if they provided an incorrect answer, they were given an opportunity to count the items and fix their response, “Can you count and make sure (*Ali lahko prešteješ in se prepričaš*)?” The following Slovenian translations were used for the Give-N task: (1) “Can you put *N* in the box?” / “*Ali lahko prestaviš* N *v škatlico*?”; (2) “Is that *N*” / “*Je to* N?”; (3) “Can you count and make sure?” / “*Ali lahko prešteješ in se prepričaš*?”

Children were asked for each number three times, in fixed pseudorandom order following the previously developed method ([7]; [26]). A subset of children failed to complete all trials. In order to maximize the amount of data collected, children who were visibly frustrated (and failing) at the large number trials were invited to complete small number trials (1, 2, and 3) in order to ensure that we collected data on knowledge of these small numbers, which were most critical to our main research questions. As noted below, all but 4 children reported here had sufficient data to assign knower levels according to established criteria (described in [11]). Critically, this approach did not introduce bias in knower level classification (e.g., favoring the detection of low knower levels), since we only classified a child as an *N*-knower given evidence that they both comprehended *N* and failed to comprehend *N*+1.

Children were classified as N-knowers for the largest number for which: (a) they provided *n* items 2/3 of the time when asked for *n*, and (b) on 2/3 trials on which they gave *n*, it was in response to a request for *n*, and not some other number [11]. In order to be classified as a non-, 1-, 2-, 3-, or 4-knower, children were required to demonstrate failure on higher numbers–so, for example, no child could be classified as a 2-knower without both demonstrating knowledge of 1 and 2 and failure on 3. Children were called non-knowers if they failed to demonstrate knowledge of the meanings of any number words, and were called Cardinal Principle knowers (CP-knowers) if they could give quantities correctly for all numbers up to and including at least 5. Three experimenters independently coded knower level for each child, and reached clear consensus on all children. Children who failed to provide data for Give-N (*n* = 6) were excluded from all analyses.

### Highest Count

All children also completed a Highest Count task. This task was used as a proxy for exposure to number language. Children were asked to count as high as they could. Children who did not understand the request or who did not begin counting were prompted by the experimenter with “one… (*ena…*)” (delivered with rising intonation). A child’s Highest Count was defined as the highest number counted to without making an error. Only one child (an English-speaker) counted to 100 or higher.

## Results

### Data Management

Children who failed to count at all (*n* = 50) were excluded from all analyses that included Highest Count as a factor, since we could not know whether their failure to count indicated a lack of knowledge of counting or a misunderstanding of the task. In total, 73 Slovenska Bistrica children (age range: 2;1–4;11; *M* = 3.65 years), 61 Ljubljana children (age range: 2;1–4;9 months; *M* = 3.25 years), 63 Metlika (age range: 2;0–4;11; *M* = 3.69 years) children, 68 Nova Gorica children (age range: 2;3–4;11.; *M* = 3.64 years), and 70 English-speaking children (age range: 2;1 to 4;11 months; *M* = 3.57 years) contributed to all analyses involving knower level and highest count.

### Counting

**Fig 2:**
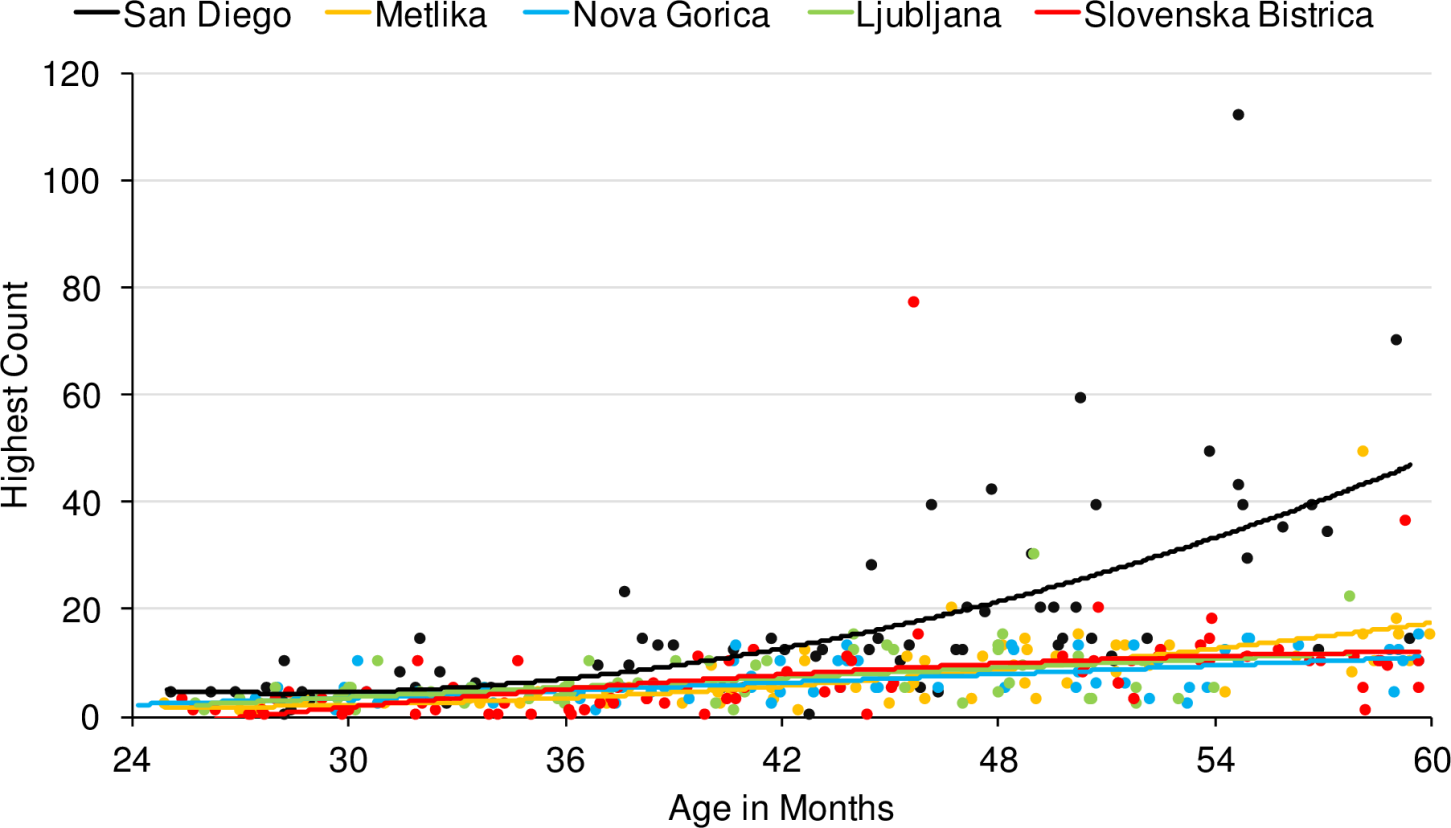
Highest count data for San Diego, Metlika, Nova Gorica, Ljubljana, and Slovenska Bistrica groups.

As noted above, the Highest Count measure served as a proxy for exposure to number language. We report these data first because Highest Count is included below as a predictor in our analyses of the Give-N data. To test whether our samples differed with respect to counting ability, we constructed a model predicting Highest Count from Language Group, Age, and an Age*Language Group interaction. Unsurprisingly, we found that Age significantly predicted Highest Count (*B* = .50, *SE* = .06, *p* < .0001). Also, Language Group was a significant predictor of Highest Count (*B* = -2.92, *SE* = 1.12 *p* = .01), and there was a significant interaction of Age and Language Group (*B* = -.28, *SE* = .12, *p* = .021). As can be seen in Fig 2, English-speaking children from San Diego counted higher (*M* = 17.90, *SD* = 18.54) than Slovenian-speaking children who spoke the Ljubljana dual dialect (*M* = 6.49, *SD* = 5.27), the Slovenska Bistrica dual dialect (*M* = 8.04; *SD* = 9.93), the Nova Gorica non-dual dialect (*M* = 6.84, *SD* = 3.97) and also the Metlika non-dual dialect (*M* = 7.83, *SD* = 7.14). Follow-up tests revealed that when only Slovenian dialects were considered, there were no significant differences in counting ability (Age: *B* = .35, *SE* = .04, *p* < .0001; Language: *B* = .69, *SE* = .78, *p* = .37; Age*Language: *B* = -.02, *SE* = .08, *p* = 0.799).

### Give-N

Our primary hypothesis is that if singular and dual morphology encode content similar to that encoded by the number words for 1 and 2, then hearing these number words used with singular and dual marking should speed their acquisition. To test this possibility, we asked: (1) whether children learning a dual dialect were faster to become 2-knowers than children learning a non-dual dialect of the same language, (2) whether, if such a difference exists, it is specific to becoming a 2-knower, and (3) whether there are consequently more 2-knowers among children learning a dual dialect (because they are faster to get to 2, but not especially fast to learn higher numbers).

We conducted preliminary analyses revealing no differences between non-dual dialects on any of our critical analyses (all *p* >.10). Also, as we discuss below, our preliminary analyses revealed only one analysis (the frequency on knowing at least 2) in which the two dual dialects differed from one another. For all analyses, we first compared the frequency of N-knowers within the Slovenian dual dialects to the frequency within the Slovenian non-dual dialects, while controlling for counting ability and age. Second, we predicted the frequency of children who knew “at least 2” from an expanded Language Group term (non-dual vs. Ljubljana vs. Slovenska Bistrica) in order to test whether our two dual dialects independently differed from our non-dual dialects. Due to the large number of children who failed to count at all (and who were necessarily excluded from the analyses that used counting as a predictor), we ran an additional set of post-hoc analyses on *all* children, removing counting as a predictor. The results of these analyses did not differ from those reported below.

**Fig 3:**
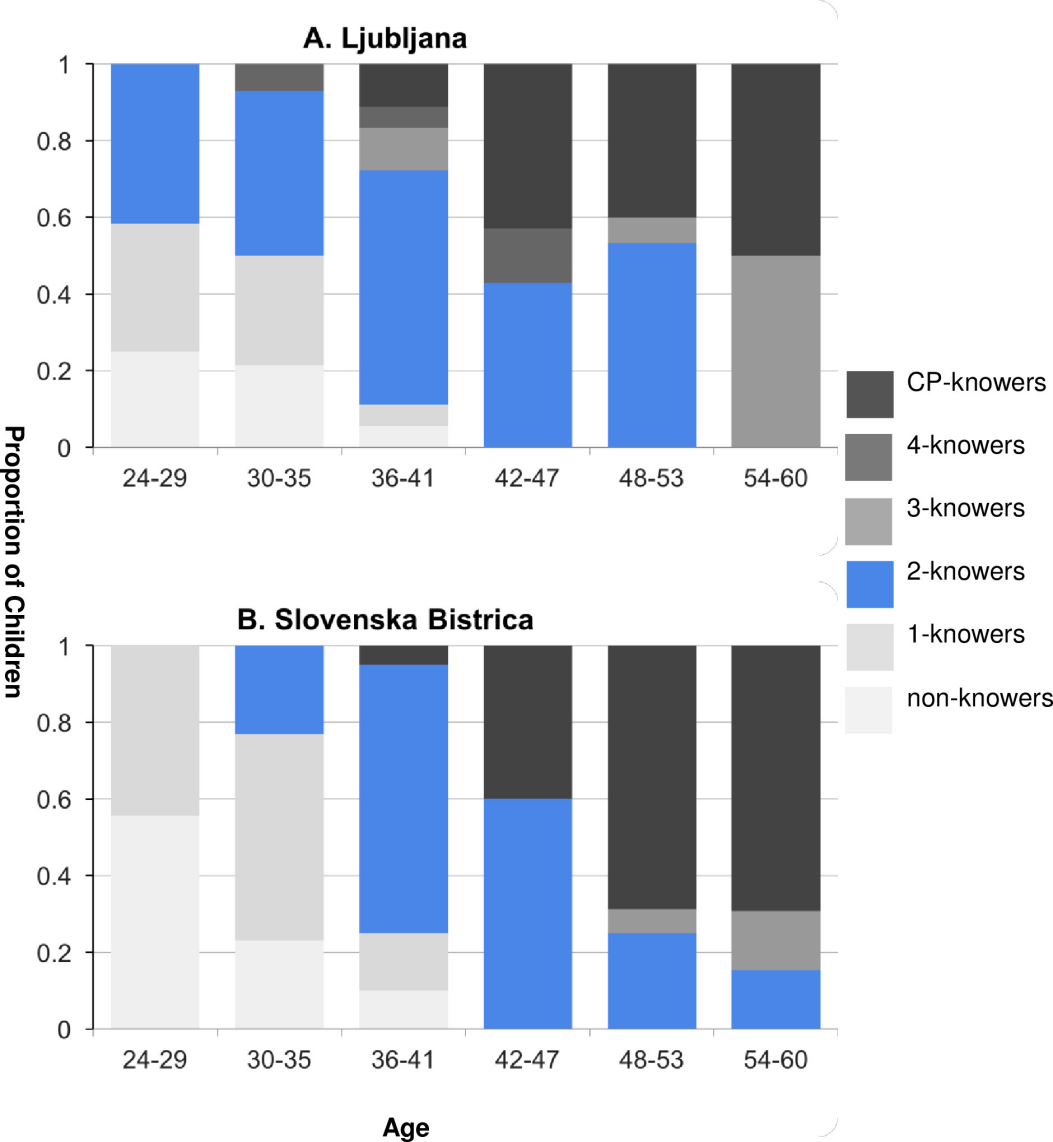
Proportion of dual learning children classified as non-knowers, 1-knowers, 2-knowers, 3-knowers, 4-knowers, and CP-knowers between 22 and 60 months of age in (A) Ljubljana and (B) Slovenska Bistrica.

**Fig 4:**
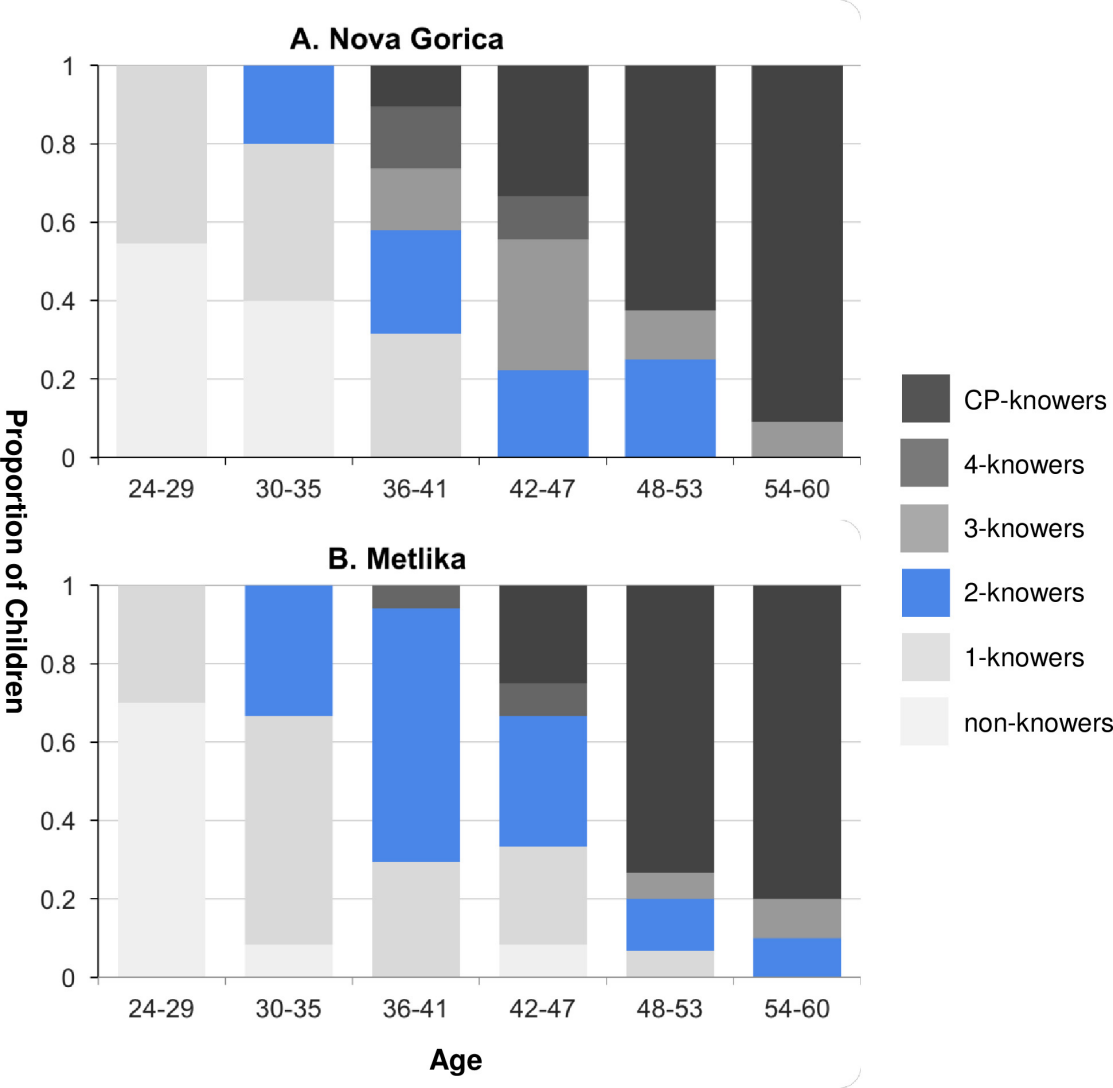
Proportion of non-dual learning children classified as non-knowers, 1-knowers, 2-knowers, 3-knowers, 4-knowers, and CP-knowers between 22 and 60 months of age in Nova Gorica and Metlika.

In our first analysis, we compared the frequency of 2-knowers among learners of the Slovenian dual dialects (Ljubljana, Slovenska Bistrica) to the frequency among learners of non-dual dialects (Metlika, Nova Gorica). Data for dual learning children are presented in Fig 3. Data for non-dual learning children are presented in Fig 4. Overall, 22.9% of children learning non-dual dialects were 2-knowers (Metlika = 29%; Nova Gorica = 18%), while 41% of children were 2-knowers in the dual dialects (Ljubljana = 49%; Slovenska Bistrica = 34%). As noted above, these numbers did not differ substantially when children who lacked Highest Count data were included (Metlika: 31%; Nova Gorica: 15%; Ljubljana: 43%; Slovenska Bistrica: 50%). We constructed a model predicting the likelihood of being a 2-knower from Language Group, Highest Count, and Age, and found an effect of Language Group (*B* = .89, *SE* = .28, *p* = .002), an effect of counting (*B* = -.16, *SE* = .04, *p* = .0003), and no effect of Age (*B* = .03, *SE* = .19, *p* = .13). Overall, speakers of dual dialects were more likely to be 2-knowers than speakers of non-dual dialects. We next asked whether each dual dialect differed from the Non-Dual dialects in frequency of 2-knowers, by predicting the likelihood of being a 2-knower from Location (Ljubljana vs. Slovenska Bistrica vs. both Non-Dual dialects), Highest Count, and Age. Non-dual dialects differed from Ljubljana (*B* = -1.26, *SE* = .35, *p* = .0003), but not from Slovenska Bistrica (*B* = .58, *SE =* .33, *p* = .08). As previously noted, our preliminary analyses revealed that children from Slovenska Bistrica did not differ significantly from the Ljubljana children in this analysis, either (*B* = -.68, *SE* = .38, *p* = .07). However, the raw numbers of 2-knowers in each group suggest a trend towards a difference in 2-knower frequency between the Ljubljana and Slovenska Bistrica groups, and between the Non-Dual and Slovenska Bistrica groups. Thus, 2-knower frequency among children from Slovenska Bistrica was somewhere between the frequencies found for Ljubljana and the Non-Dual learners.

These first analyses showed that 2-knowers were more frequent in the dual dialects of Slovenian than in the two non-dual dialects, and that this effect was driven more by children in the Ljubljana sample than by children from Slovenska Bistrica. We next asked whether children learning dual dialects were *faster* to become 2-knowers than children learning non-dual dialects. To assess this, we compared the frequency of non- and 1-knowers to the frequency of children who knew the meaning of *at least* 2 (i.e., the frequency of 2-, 3-, 4- and CP-knowers) across languages. We constructed models predicting knowledge of 2*+* from Age, Language Group, and Highest Count. We found an effect of Language Group (*B* = 1.12, *SE* = .42, *p* = .008), an effect of counting (*B* = .23, *SE* = .09, *p* = .01), and an effect of Age (*B* = .24, *SE* = .04, *p* < .0001). Speakers of the dual dialects showed a modest but significant difference in their likelihood of being at least 2-knowers (76.12% in dual dialects vs. 70.99% in non-dual dialects). We next asked whether each dialect differed from Ljubljana in frequency of 2+ knowers, by predicting the likelihood of being a 2-knower or higher from Location (Ljubljana vs. Slovenska Bistrica vs. all Non-Dual dialects), Highest Count, and Age. Children from Ljubljana (80% 2+ knowers) differed from those learning non-dual dialects (71% 2+ knowers; *B* = -1.82, *SE* = .54, *p* = .0008), while children in Slovenska Bistrica did not differ from those learning non-dual dialects (73% 2+ knowers; *B* = .47, *SE* = .50, *p* = .35). Recall that preliminary analyses found that children in Slovenska Bistrica differed from children in Ljubljana (*B* = 1.52, *SE* = .65, *p* = .02). Thus, while children learning dual dialects were generally faster to become 2-knowers than children learning non-dual dialects, children in Ljubljana were fastest among all four groups.

We next tested whether acquiring a dual dialect confers a learning benefit for numbers larger than 2. If learning the dual specifically facilitates learning the meaning of 2, then we would expect no facilitative effect of the dual on learning the meanings of larger numbers, like 3. Instead, other factors like counting experience should explain differences in knowledge of 3 (as reported by [7]). To test this, we classified children as either knowing at least 3 (3-, 4-, and CP-knowers) or not (non-, 1-, and 2-knowers). Overall, 41% of children from Metlika and 54% of children from Nova Gorica were 3+ knowers, while 3+ knowers made up 31% of children in the sample of Ljubljana children and 38% in Slovenska Bistrica children. We asked whether Age, Highest Count, and Language Group predicted being a 3+ knower. We found a marginally significant effect of Language Group (*B* = -.73, *SE* = .38, *p* = .055), as well as significant effects of counting (*B* = .30, *SE* = .05, *p* < .0001), and Age (*B* = .15, *SE* = .03, *p* < .0001). We next predicted the likelihood of being a 3+ knower from Location (Ljubljana vs. Slovenska Bistrica vs. both Non-Dual dialects), Highest Count, and Age. The Ljubljana dialect did not differ from the Non-Dual dialects (*B* = -.40, *SE* = .47, *p* = .39), although the Slovenska Bistrica dialect did (*B* = -1.04, *SE* = .47, *p* = .03). Together, these results indicate that speakers of dual dialects were no more likely to know the meaning of 3+ than were speakers of non-dual dialects; in fact, participants from Slovenska Bistrica were *less* likely to be 3+ knowers than their non-dual counterparts.

### Give-M

The Give-M task measured the degree to which children from the four Slovenian speaking samples understood the singular, dual, and plural forms, and thus allowed us to test how learning these forms relates to number word learning. To do this, we computed the mean percentage of correct responses for each child for each morphological ending (singular, dual, plural) separately. We conducted two sets of analyses. First, we asked whether children in each group comprehended each form. Next, we asked how this knowledge was related to knower level developmentally.

We first present data from singular and plural trials. Children from all samples exhibited strong knowledge of the singular (*M*_Ljubljana_ = 80.42%; *M*_Slovenska Bistrica_ = 62.55%; *M*_Nova Gorica_ = 82.10%; *M*_Metlika_ = 55.29%), as measured by their ability to give exactly one object (correct performance was giving 1 out of 10 objects). Also, speakers of all four Language Groups performed well on plural trials, though the prior likelihood of a correct response was quite high, since it was defined as giving between 3–10 objects out of 10 possible objects (*M*_Ljubljana_ = 81.87%; *M*_Slovenska Bistrica_ = 88.86%; *M*_Nova Gorica_ = 80.55%; *M*_Metlika_ = 82.25%). Together, these data suggest that children understood the task, and children in our samples provided sensible responses for singular and plural trials.

Next, we considered performance on the dual trials, where correct performance was defined as providing exactly 2 objects. Again, children in all samples performed well (*M*_Ljubljana_ = 55.29%; *M*_Slovenska Bistrica_ = 46.08%; *M*_Nova Gorica_ = 50.51%; *M*_Metlika_ = 25%). Thus, not only did children appear to understand the task, but children in all samples exhibited some knowledge of the dual. This suggests that, even in regions where the dual is not generally used, many children nevertheless receive enough exposure to the form (e.g. through some inevitable contact with Standard Slovenian) to eventually comprehend the form, though as we report below, they acquire it later. Also, as shown by a follow-up study conducted in Nova Gorica, while children learning non-dual dialects eventually comprehend the dual, they do not hear it enough to acquire it as part of their spoken language, and do not use it spontaneously when labeling quantities.

We conducted an additional, follow-up study of 39 young (24–35 months old; *M* = 31.07 months; only 5 of these children, or 12.8%, were 2-knowers) children in Nova Gorica, in order to further assess knowledge of the dual in this sample. Specifically, we tested children using the What’s on this Card task to test whether they would spontaneously use the dual when labeling sets of two. We found that children from Nova Gorica did so on only 8.63% of dual trials, which was far less often than age-matched children (*N =* 32; Age = 24–36 months; *M* = 31.61 months) learning Central Slovenian in Ljubljana [7], who did so 47.14% of the time. This finding suggests that dual knowledge may contribute to number word learning even in non-dual dialects, though probably not to the same degree as children who receive input that consistently includes the dual and who ultimately acquire the dual as a part of their native dialect.

The critical question of our study is how the acquisition of dual morphology is related to number word learning in each group. To explore this question, we asked whether there was a substantial increase in dual knowledge that occurred around the time that a child mastered the meaning of the word for 2. We predicted that this correlation should be present among children who consistently hear number words used in dual morphology. As previously reported [7], children learning Central Slovenian in Ljubljana demonstrated much higher rates of dual knowledge at the 2-knower stage (*M* = 61.2% correct on dual trials), than at the non-knower and 1-knower stages (combined *M* = 21.9% correct on dual trials; Wilcoxon *p* = .002). However, there was no corresponding difference in dual knowledge between 2-knowers and 3+ knowers (*M* = 75.5% correct on dual trials; relative to 2-knowers Wilcoxon *p* = .37). Consistent with this, we found that children in the Slovenska Bistrica also had greater knowledge of the dual at the 2-knower stage (*M* = 50.86% correct) relative to the non-knower and 1-knower stages (combined *M* = 8.33% correct; *p* < .0001). However, they showed a much smaller increase in dual knowledge when going from knowing 2 to knowing 3+ (*M* = 75%, *p* = .006). In contrast, children in the Metlika and Nova Gorica samples showed no relation between dual knowledge and knower level: Knowledge of the dual was not different between 2-knowers and children with lower knower levels (Nova Gorica: *p* = .12; Metlika: *p* = .31), though there was a difference between 2-knowers and 3+ knowers (Nova Gorica: *p* = .0002; Metlika: *p* = .0006). This suggests that while some of the children in our non-dual dialect samples eventually gained familiarity with the dual, this learning appeared to occur later in development–largely *after* these children learned the meaning of *three*.

### Comparison to English

Our analyses thus far have compared the dual and non-dual dialects of Slovenian and have asked: (1) How number word learning differed across dialects, and (2) How learning the dual morphology was related to number knowledge within each dialect. The main premise of this study was that comparing across dialects within Slovenia would provide a stronger test of the hypothesis that dual morphology, *per se*, explains differences between children’s rate of number word acquisition. Thus far, we have found evidence that, overall, dual learners are faster to become 2-knowers, but also that there is some variability across dialects with respect to the strength of this effect. Also, consistent with the idea that morphological knowledge drives such differences, we found that in dual dialects only, knower level is correlated with children’s comprehension of dual morphology.

While these comparisons provide the strongest test of how morphology affects learning, it is nevertheless useful to compare these data to other findings in the literature (e.g., from English, the most studied language group) to establish whether morphological effects are detectable when placed in the context of other cross-cultural differences. For example, young English-speaking children in the US appear to receive substantially more training in the counting routine than any of the groups studied in Slovenia (see Fig 2), and in our study were sampled from a uniformly high SES urban environment. The Slovenian groups, however, varied with respect to the education and income levels of the populations from which they were sampled, perhaps in part explaining differences between groups within the country (see Fig 1 and Table 1).

In our previous study we compared data from Ljubljana to data from English-speaking children in the US, and found that the Slovenian dual learners were both more likely to be 2-knowers and more likely to know at least 2 relative to children in the US (and relative to Japanese-, Mandarin- and Russian-speaking children from Japan, Taiwan, and Russia). Thus, for comparison, we asked here whether children from Slovenska Bistrica also differed from US English-speaking children. This analysis found that children from Slovenska Bistrica were marginally more likely to be 2-knowers than children in the US (Slovenska Bistrica: 34%; English: 12%; *B* = .92, *SE* = .51, *p* = .07). However, it also found that they were no more likely to know at least 2 (English: 68%; Slovenska Bistrica: 73%; *B* = 1.2, *SE* = .78, *p* = .12). Thus, although the children from Slovenska Bistrica exhibited the same qualitative pattern of difference from English children as observed when considering Ljubljana children, the effects did not reach statistical significance. We revisit this result in the General Discussion, and consider why effects of grammatical variation found within a culture may not be found when comparing cross-culturally.

## General Discussion

We investigated number word learning in children learning two non-dual dialects of Slovenian, and compared them to children learning two Slovenian dialects in which the dual is habitually used. Our goal was to test the hypothesis that cross-linguistic differences in the morphological marking of number are related to differences between cultures in the acquisition of number word meanings, and, by extension, that these different systems of number representation draw on a shared conceptual hypothesis space ([5]; [7]; [24]; [25]; [26]; [28]).

We found that among children acquiring the same language, learning the meaning of the word for 2 differed according to regional variation in the use of dual morphology. Although many children learning all four dialects eventually came to comprehend the dual, those exposed to non-dual dialects appeared to comprehend it later in development, and did not hear it frequently enough that it was adopted in their own spontaneous speech. Whereas children from Ljubljana spontaneously use the dual when labeling sets in the “What’s on this Card” task [7], we found that children from Nova Gorica almost never use the dual in this task. Corresponding to this, children exposed to non-dual dialects–who therefore learned to comprehend the dual late or not at all–were slower to become 2-knowers relative to children exposed to dual dialects, who learned the dual early in life. Also, children learning non-dual dialects not only became 2-knowers later, but 2-knowers were overall less frequent among these children, relative to children learning dual dialects. Finally, we found that learning the dual, as measured by the Give-M task, was related to becoming a 2-knower, but only among children exposed to a dual dialect: Children from Ljubljana and Slovenska Bistrica who comprehended the dual were significantly more likely to know the meaning of the word for 2 than those who did not. The same was not true among children learning non-dual dialects, who appeared to learn the dual sometime after becoming 2-knowers.

These data are consistent with the hypothesis that, when concepts like singular and dual are explicitly marked in a language, children are faster to acquire corresponding number words. Because the Slovenian children in our study lived in the same country, were culturally similar, spoke almost identical languages, and had almost identical counting abilities (as measured by Highest Count), the likelihood that differences in number word learning could be explained by unmeasured cultural differences is much smaller here than in previous work. For example, in some past studies it was reported that English-speaking children in the US became 1-knowers earlier than both Japanese- and Mandarin-speaking children in Japan ([25]; [26]) and Taiwan [24]. Also, differences in knowledge of the word for 2 were reported between dual learners in Saudi Arabia and Slovenia compared to children in other language groups [7]. In each case, however, linguistic differences covaried with substantial cultural differences, leaving open the possibility that unmeasured factors explained variability in number word learning. Here, we minimized the likelihood of such extraneous non-linguistic differences, and found that children learning non-dual dialects of Slovenian were slower to become 2-knowers compared to children learning dual dialects. In support of this general approach, we also showed that effects due to morphology which are found within a culture may not be detectable when comparing groups across different cultures like the US and Slovenia–e.g., if children differ in other unmeasured ways like counting ability, parental education, parental income, etc., that can potentially swamp linguistic differences. Similarly, we found that fairly substantial variability can exist between groups from the same country even when their grammars do not differ with respect to number morphology, again, perhaps owing to secondary factors (e.g., the higher income and education levels of families in Ljubljana relative to those of families in Slovenska Bistrica). These findings suggest that randomly sampling groups from two different countries to assess effects of language on cognitive development may increase the risk of both false positive and false negative effects, and that such comparisons should be avoided if possible, or complemented with extensive batteries of tasks that can be used to match children cross-culturally [29] (see [30] for a more general discussion about spurious correlations).

The relationship that we report between number word learning and the acquisition of number morphology may be important to understanding the nature of children’s first number word meanings, even outside of languages like Slovenian. First, the correlation suggests that the two systems encode similar conceptual content: Learning the singular likely helps children become 1-knowers because both forms encode singleton sets, just as learning the dual facilitates becoming a 2-knower because both encode dual sets. If this is right, then learning concepts like 1, 2, and 3 may not constitute an instance of domain-specific conceptual change that is special to human mathematical cognition. Instead, these concepts may be more general, and foundational to systems of natural language morphology. On this view, young children learning words for 1, 2, and 3 may deploy the same conceptual resources as when they learn singular, dual, and trial forms (for discussion of the nature of this conceptual hypothesis space, see [8]). This hypothesis would not only explain the developmental correlations we report here, but also the striking typological observation that, just as children cannot learn the meanings of words higher than 4 in absence of counting, some natural languages also feature grammatical markers of singular, dual, and trial, but not of precise sets of four of more ([14]; for discussion, see [6]; [17]; [31]). From the perspective of the child, these systems may be semantically identical in nature.

Another implication of this work is that the use of number words with informative linguistic cues, like number morphology, may be as important as counting routines to early number word learning even in languages that do not feature dual morphology. Our data, and data from other studies ([24]; [25]; [26]), suggest that when number words are used in conversation with informative grammatical cues, they can be learned despite relatively low frequency and little to no formal training with counting procedures. In each of the Slovenian samples tested, children were barely able to count past their knower levels, indicating very little exposure to number words and counting in their input. Nevertheless, they were more likely to be 2-knowers when their dialect featured dual morphology and they comprehended the dual. Similarly, there is also evidence, though weaker for reasons just discussed, that English-speaking children become 1-knowers before children learning Japanese and Mandarin due to the presence of singular-plural morphology in English, and that this difference arises despite the fact that mathematics training is equally or more intensive in these cultures, relative to the US ([32]; [33]; [34]). Although some languages provide sparser cues than others, most nevertheless provide multiple grammatical cues to indicate that number words encode number ([25]; [28]; [35]; [36]). Such cues–like quantifiers, classifiers, and optional plural forms could also provide children a rich basis for narrowing the meanings of their first few number words.

In sum, we have presented evidence that morphological marking of number in language facilitates learning of early number word meanings. We compared children learning four dialects of Slovenian and found that, overall, dual learners were faster than non-dual learners to become 2-knowers, when controlling for factors like age and counting ability, and that comprehension of the dual was related to number knowledge in children learning dual dialects, but not in children learning non-dual dialects. Although these children ultimately learned to comprehend the dual–which may have contributed modestly to their becoming 2-knowers–this generally happened after they became 2-knowers, and was not sufficiently robust as to be reflected in their spontaneous speech, which lacked dual usage almost completely. Together, these results support the idea that number word learning likely relies on content similar to that used for acquiring number morphology, and thus that mathematical knowledge may be built on a foundation of concepts that arise spontaneously in natural language, even in absence of formal training with number.

## Supporting Information

S1 File Supporting Information. This document describes the linguistic and Socio-economic differences between the four locations where children were tested in Slovenia.
(PDF)
