# Supplementary material for: Does Grammatical Structure Accelerate Number Word Learning? Evidence from Learners of Dual and Non-Dual Dialects of Slovenian
Source: PLoS One. 2016 Aug 3;11(8):e0159208. doi: 10.1371/journal.pone.0159208 (PMC4972304; doi:10.1371/journal.pone.0159208)
Supplement: S1 File — This document describes the linguistic and Socio-economic differences between the four locations where children were tested in Slovenia. (PDF) [file pone.0159208.s001.pdf]

Supporting Information (for the paper Franc Marušič, Rok Žaucer, Vesna Plesničar, Tina Razboršek, Jessica Sullivan, & David Barner: "Language as a source of small number concepts: Evidence from dual and non-dual learners of Slovenian")

This document describes the linguistic and Socio-economic differences between the four locations where children were tested in Slovenia.

Linguistic Background

Slovenian is a South-Slavic language whose grammatical number system distinguishes between singular, dual, and plural (1-3) in nouns, pronouns, adjectives, and verbs. That is, whereas singular-plural languages such as English morphologically distinguish between 'red button' (used in reference to one) and 'red buttons' (used in reference to more than one), Slovenian number morphology distinguishes distinct forms of a single noun that will be used in reference to one item (singular), in reference to two items (dual), and in reference to more than two items (plural). In addition, when a noun is modified by an adjective (as in 'red button'), the adjective agrees with the noun, so that adjectives also show forms that are specifically singular, dual and plural. This is shown in Table S1, which lists the forms for all six cases.

Table S1: Declension paradigms for inanimate masculine nouns of declension class I (*gumb* 'button'), for inanimate-masculine-noun-modifying adjectives (*rdeč* 'red'), and for inanimate-masculine-noun-modifying small number words (*one*, *two*, *three/four*) in Standard Slovenian

	Singular			Dual			Plural		
Case	Num	Adj	N	Num	Adj	N	Num	Adj	N
Nominative	*en*	*rdeč*	*gumb*	*dva*	*rdeča*	*gumba*	*trije*	*rdeči*	*gumbi*
Genitive	*enega*	*rdečega*	*gumba*	*dveh*	*rdečih*	*gumbov*	*treh*	*rdečih*	*gumbov*
Dative	*enemu*	*rdečemu*	*gumbu*	*dvema*	*rdečima*	*gumboma*	*trem*	*rdečim*	*gumbom*
Accusative	*en*	*rdeč*	*gumb*	*dva*	*rdeča*	*gumba*	*tri*	*rdeče*	*gumbe*
Locative	*enem*	*rdečem*	*gumbu*	*dveh*	*rdečih*	*gumbih*	*treh*	*rdečih*	*gumbih*
Instrumental	*enim*	*rdečim*	*gumbom*	*dvema*	*rdečima*	*gumboma*	*tremi*	*rdečimi*	*gumbi*
	'one red button'			'two red buttons'			'three red buttons'		

Although there is some syncretism (i.e., morphological overlap) between the dual and plural forms of both nouns and adjectives (in the case of masculine nouns of declension class I, this holds in two of the six cases, as seen in Table S2, and similarly for feminine and neuter nouns), the dual and the plural are nevertheless clearly distinct categories with overall distinct morphological marking. Furthermore, through agreement with the subject, distinct singular/dual/plural number marking also surfaces on the verb, as shown in Table S2 for the present indicative and imperative paradigms.

Table S2: Present indicative and imperative conjugation paradigms (*držati* 'hold') in Standard Slovenian

Mood	Number	Person 1	Person 2	Person 3
Indicative	Singular	*drži**m***	*drži**š***	*drží*
	Dual	*drži**va***	*drži**ta***	*drži**ta***
	Plural	*drži**mo***	*drži**te***	*drži**jo***
Imperative	Singular	/	*dŕži*	/
	Dual	*drži**va***	*drži**ta***	/
	Plural	*drži**mo***	*drži**te***	/

The robustness of the dual is greatest in the standard variety, which is the variety used for writing, taught at schools, used in mainstream media and by some speakers in formal situations. Across dialects, however, the robustness of the dual varies considerably – see map S1, which is based on fieldwork data from (4) and shows the ratio of all special dual forms reported for any sampled location out of the maximally possible amount of special dual forms.

Map S1: **Dual in Slovenian dialects – quantified**[1]

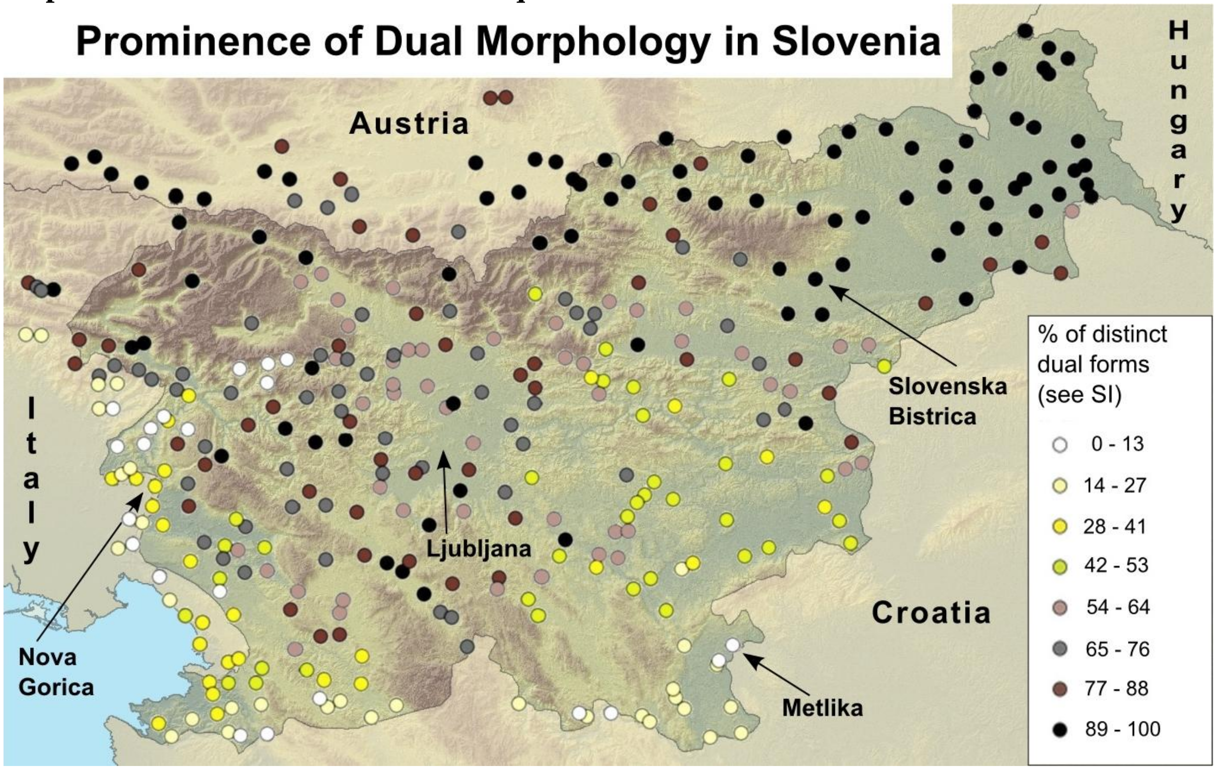

In what follows, we outline a subset of the relevant aspects of the varieties of Slovenian spoken in the three towns where we tested: in Ljubljana, located roughly in the center of Slovenia and the Slovenian-speaking territory; in Nova Gorica, located on the border with Italy, at the western periphery of the Slovenian-speaking territory; in Slovenska Bistrica, centrally located in the eastern part of Slovenia; and in Metlika, located on the border with Croatia, at the south-eastern periphery of the Slovenian-speaking territory (see map S1). The dialects of Ljubljana and

---

[1] **Map S1** is based on fieldwork data from (4) and shows the ratio of all special dual forms reported for any sampled location out of the maximally possible amount of special dual forms. It includes those sampled locations for which data is reported for at least 10 grammatical contexts (maximum is 26 grammatical contexts), yielding a total of 360 plotted locations. (4) provides data for a further 25 locations with forms reported for anywhere between 1 and 9 grammatical contexts. To quantify the presence of special dual marking for each included location, we summed up the dual forms for the reported grammatical contexts as follows: the “semantically dual” grammatical contexts for which the use of only dual forms is reported were counted as 1, those for which the use of only plural forms is reported were counted as 0, and those for which variation between the dual and plural forms is reported were counted as partially dual, as 0.5. This method is further discussed below. The background for this map is from Wikimedia (https://upload.wikimedia.org/wikipedia/commons/3/37/Relief_map_of_Slovenia.png; created by Geologicharka (Own work) [CC BY-SA 3.0 (http://creativecommons.org/licenses/by-sa/3.0)], via Wikimedia Commons).

Slovenska Bistrica generally exhibits more robust dual marking than the dialects of Nova Gorica and Metlika (4).

The most prominent difference between the Ljubljana and Slovenska Bistrica varieties on the one hand and the varieties of Nova Gorica and Metlika on the other is number agreement on the verb. When a nominative subject refers to a set of two, Ljubljana and Slovenska Bistrica Slovenian use a verb form that is distinct from the form used when the subject refers to a set of more than two. This holds regardless of whether the noun in the subject position belongs to the group with distinct dual and plural forms or to the group with syncretic dual and plural forms, and it also holds when the subject is a null pronoun. The varieties of Nova Gorica and Metlika, however, generally use a single verb form regardless of whether the subject (overt or null) denotes a set of two or of more than two, and this is the verb form that corresponds to the plural verb form in the variety of Ljubljana (4) (see Table S3).[2]

Table S3: Present indicative conjugation paradigms for first-person subjects in singular/dual/plural for Ljubljana, Slovenska Bistrica, Nova Gorica and Metlika.[3]

Subject set	one	two	three
LJ	*Delam.*	*Delava.*	*Delamo.*
	work.SG	work.DU	work.PL
SB	*Delam.*	*Delama.*	*Delamo.*
	work.SG	work.DU	work.PL
NG	*Delam.*	*Delamo.*	*Delamo.*
	work.SG	work.PL	work.PL
ME	*Delam.*	*Delamo.*	*Delamo.*
	work.SG	work.PL	work.PL
	'I am working'	'We are working.'	'We are working'

Jakop (2008) reports the forms that are used in various Slovenian dialects in a “semantically dual” context for 31 combinations of specific lexical items and grammatical contexts (4). She reports, for example, the actual forms used in various dialects when the consonant-stem masculine noun ‘son’ occurs in nominative case in a “semantically dual” context, and codes them as dual-morphology forms or plural-morphology forms. Table (S4) provides a complete list of those 31 forms for the three locations at which we conducted our tests: for Ljubljana (reported are aggregate data for two Ljubljana-suburbs sampling points: point 249-Rudnik - Ljubljana and 250-Studenec - Ljubljana); for Nova Gorica (reported are aggregate data for three Nova Gorica-adjacent sampling points: 96-Grgar, 97-Solkan, 100-Šempeter pri Gorici); and for Metlika (reported are aggregate data for four Metlika-adjacent sampling points: 293-Vavpča vas, 294-Podzemelj, 295-Grm pri Podzemlju, 296-Metlika).

---

[2] The situation is as described above when a nominative subject includes no element with overt reference to the size of the set or when a nominative subject includes an overt numeral ‘one’, ‘two’, ‘three’ or ‘four’ (which behave like adjectives). When the subject contains an overt quantifier like ‘many’ or an overt numeral ‘five’ or higher (which behave like quantifiers), the subject’s head noun receives quantificational/genitive plural marking, which fails to trigger agreement, so the verb receives default, i.e. 3rd person singular, agreement in all varieties of Slovenian (see 5 & 6 for details).

[3] The data for subject sets of two is based on (4) and given in standardized spelling.

Table S4: Comparison of dual-form use in Slovenska Bistrica, Ljubljana, Nova Gorica and Metlika according to Jakop (2008) (N = Noun, Adj. = Adjective, Num. = Numeral, Pron. = Pronoun, V = Verb, M = Masculine, F = Feminine, N = Neuter, Nom. = Nominative, Dat. = Dative, Du = Dual, Pl = Plural, P = Person, Pres.T = Present tense)

	I	II	III	IV	V
	Standard Slovenian form **Form description “translation”**	***Slovenska Bistrica form*** **(form description)**	***Ljubljana form*** **(form description)**	***Nova Gorica form*** **(form description)**	***Metlika form*** **(form description)**
1	*sin(ov)a* N M, C-stem, Nom.Du “(two) sons”	no data	*sinova* (Nom.Du)	*sina* (Nom.Du)	*sina / sini* (Nom.Du)
2	*sin(ov)oma* N M, C-stem, Dat.Du “to (two) sons”	no data	*sinovoma/sinovom* (Dat.Du / Dat.Pl)	*sinovom* (Dat.Pl)	*sinom* (Dat.Pl)
3	*brata* N M, O-stem, Nom.Du “(two) brothers”	*brata* (Nom.Du)	*brata* (nom.Du)	*brata* (Nom.Du)	*brata* (Nom.Du)
4	*bratoma* N M, O-stem, Dat.Du “to (two) brothers”	*bratoma* (Dat.Du)	*bratoma/bratom* (Dat.Du / Dat.Pl)	*bratom* (Dat.Pl)	*bratom* (Dat.Pl)
5	*kravi* N F, A-stem, Nom.Du “(two) cows”	*kravi* (Nom.Du)	*krave* (Nom.Pl)	*kravi* (Nom.Du)	*kravi / krave* (Nom.Du / Nom.Pl)
6	*kravama* N F, A-stem, Dat.Du “to (two) cows”	*kravama* (Dat.Du)	*kravama/kravam* (Dat.du / Dat.Pl)	*kravam* (Dat.Pl)	*kravam* (Dat.Pl)
7	*hčeri* N F, C-stem, Nom.Du “(two) daughters”	*hčeri* (Nom.Du)	*hčere* (Nom.Pl)	*hčeri* (Nom.Du)	*hčeri / hčere* (Nom.Du / Nom.Pl)
8	*hčerama* N F, C-stem, Dat.Du “to (two) daughters”	*hčerama* (Dat.Du)	*hčerama/hčeram* (Dat.du / Dat.Pl)	*hčeram* (Dat.Pl)	*hčeram* (Dat.Pl)
9	*okni* N N, O-stem, Nom.Du “(two) windows”	*okni* (Fem.Nom.Du)	*okna* (Masc.Nom.Du)	*okni* (Neut.Nom.Du)	no data
10	*midva* Pron. M, 1st P, Nom.Du “two of us”	*midva* (Nom.Du)	*midva/medva* (Nom.Du / Nom.Du)	*midva/medva/mi* (Nom.Du/Nom.Du /Nom.Pl)	*midva / mi* (Nom.Du / Nom.Pl)
11	*medve* Pron. F, 1st P, Nom.Du “two of us”	*midve/midvi* (Nom.Du)	*medve/midve* (Nom.Du / Nom.Du)	*medve/midve/me* (Nom.Du/Nom.Du /Nom.Pl)	*midve / midvi* (Nom.Du / Nom.Du)
12	*naju* Pron. M, 1st P, Gen.Du “of (two) of us”	*naju* (Gen.Du)	*naju* (Gen.Du)	*nas dveh/nas* (Gen.Pl / Gen.Pl)	*nas* (Gen.Pl)

13	*nama*<br>Pron. M, 1st P, Dat.Du<br>"to (two) of us"	*nama*<br>(Dat.Du)	*nama*<br>(Dat.Du)	*nam dvem / nam*<br>(Dat.Pl / Dat.Pl)	*nam*<br>(Dat.Pl)
14	*dobra*<br>Adj. M, Nom.Du<br>"good"	*dobra*<br>(Nom.Du)	*dobra*<br>(Nom.Du)	*dobra*<br>(Nom.Du)	*dobri*<br>(Nom.Pl)
15	*dobri*<br>Adj. F, Nom.Du<br>"good"	no data	*dobre*<br>(Nom.Pl)	*dobri*<br>(Nom.Du)	*dobre*<br>(Nom.Pl)
16	*dobrima*<br>Adj. M, Dat.Du<br>"good"	*dobrima*<br>(Nom.Du)	*dobrima/dobrim*<br>(Dat.Du / Dat.Pl)	*dobrim*<br>(Nom.Pl)	*dobrim*<br>(Dat.Pl)
17	*dveh*<br>Num. M/F, Gen.Pl<br>"of two"	*dveh*<br>(Gen.Pl)	*dveh*<br>(Gen.Pl)	*dveh*<br>(Gen.Pl)	*dveh*<br>(Gen.Pl)
18	*dvema*<br>Num. M/F, Dat.Du<br>"to two"	*dvema*<br>(Dat.Du)	*dvema/dvem*<br>(Dat.Du / Dat.Pl)	*dvema/dvem*<br>(Dat.Du / Dat.Pl)	*dvem*<br>(Dat.Pl)
19	*delava*<br>V M, 1st P, Du. Pres.T<br>"two of us work"	*delama*<br>(Du)	*delava*<br>(Du)	*delamo*<br>(Pl)	*delamo*<br>(Pl)
20	*delava*<br>V F, 1st P, Du. Pres.T<br>"two of us work"	*delama*<br>(Du)	*delava*<br>(Du)	*delamo*<br>(Pl)	*delamo*<br>(Pl)
21	*greva*<br>V M, 1st P, Du. Pres.T<br>"two of us go"	*grema*<br>(Du)	*greva*<br>(Du)	*gremo*<br>(Pl)	*gremo*<br>(Pl)
22	*greva*<br>V F, 1st P, Du. Pres.T<br>"two of us go"	*grema*<br>(Du)	*greva*<br>(Du)	*gremo*<br>(Pl)	*gremo*<br>(Pl)
23	*delata*<br>V M, 2nd P, Du. Pres.T<br>"two of you work"	*delata*<br>(Du)	*delata*<br>(Du)	*delata / delate*<br>(Du / Pl)	*delate*<br>(Pl)
24	*delata*<br>V F, 2nd P, Du. Pres.T<br>"two of you work"	*delata*<br>(Du)	*delata*<br>(Du)	*delate*<br>(Pl)	*delate*<br>(Pl)
25	*gresta*<br>V M, 2nd P, Du. Pres.T<br>"two of you go"	*greta*<br>(Du)	*gresta*<br>(Du)	*gresta / greste*<br>(Du / Pl)	*greste*<br>(Pl)
26	*gresta*<br>V F, 2nd P, Du. Pres.T<br>"two of you go"	*greta*<br>(Du)	*gresta*<br>(Du)	*greste*<br>(Pl)	*greste*<br>(Pl)
27	**Sum of dual-only**	22 dual-only cells	16 dual-only cells	7 dual-only cells	2 dual-only cells

	**cells**<br>**Sum of plural-only cells**<br>**Sum of dual/plural cells**	1 plural-only cells<br><br>Dual score: 22/23 = 95.65%	4 plural-only cells<br>6 dual/plural cells<br>Dual score: 19/26 = 73.07%	14 plural-only cells<br>5 dual/plural cells<br>Dual score: 9,5/26 = 36.54%	19 plural-only cells<br>4 dual/plural cells<br>Dual score: 4/25 = 16%

For clarity, Table S4 has cells with dual forms marked in green, cells with plural forms marked in red, and cells with variably used dual and plural forms marked in yellow. As the table clearly shows, the dialectal situation is not one with an opposition between varieties in which “semantically dual” contexts exclusively exhibit dual forms and varieties in which “semantically dual” contexts exclusively exhibit plural forms.

Starting from the bottom, rows 19 through 26 report the usage of dual/plural forms on verbs. Specifically, they show the present indicative 1st- and 2nd-person verb forms used with masculine and feminine subjects in the standard (column I) and in the three dialects (columns II through IV) for two frequent verbs (‘work/do’, ‘go’). The forms are reported as in (4), so that forms used with masculine and feminine subjects are always given separately, both when a gender distinction correlates with a difference in the use of number morphology (as in the case of 2nd person, see rows 25-26 and 23-24) and when there is no difference (as in the case of 1st person, see rows 21-22 and 19-20). Forms are not reported for 3rd person, for neuter-gender subjects, and for the imperative. As seen from rows 19-26/column II and III, the Slovenska Bistrica and Ljubljana dialects match the standard in requiring dual forms (=green) in all reported forms. As seen from rows 19-26/column IV, the Nova Gorica dialect variably uses dual or plural forms (=yellow) with 2nd-person masculine subjects and only uses plural forms (=red) in the other six reported forms. As seen from rows 19-26/column V, the Metlika dialect only uses plural forms (=red) in all reported forms.

Rows 14-18 report the use of dual/plural forms on adjectives. Usage is reported for the adjective ‘good’ for masculine and feminine gender for nominative case, for masculine gender dative case, and for the adjectivally declining numeral ‘two’ for masculine and feminine gender together for genitive and dative case. For ‘good’, genitive, accusative, locative and instrumental case forms are not reported for masculine and feminine gender; dative case form is not reported for feminine gender; no neuter gender forms are reported. For ‘two’, nominative, accusative, locative and instrumental case forms are not reported for masculine and feminine gender; and no neuter gender forms are reported. As seen from rows 14-18/column II, the Slovenska Bistrica dialect has dual cells in all but one of the reported contexts. As seen from rows 14-18/column III, out of the five cells, the Ljubljana dialect has one dual-only cell, two cells with variably dual or plural forms, and two cells with plural-only forms. As seen from rows 14-18/column IV, the Nova Gorica dialect has two dual-only cells, one cell with variably dual or plural forms, and two cells with plural-only forms. As seen from rows 14-18/column V, the Metlika dialect has only plural forms in all five cells.[4]

Rows 10-13 report the use of dual/plural forms on pronouns. Usage is reported for 1st person for masculine gender for nominative, genitive and dative cases, and 1st person for feminine gender for nominative. Forms are not reported for 1st person for masculine gender for accusative, locative and instrumental cases; for 1st person for feminine gender for genitive,

[4] The standard variety's form *dveh* "of two" in row 17 is coded as 'Num. M/F, Gen.Pl'. The genitive case ending on this form is syncretic with the genitive case ending for plural and is thus coded as plural even in the standard variety. There are dialects however, where the form in this row would have a dual ending that is different from the plural ending.

dative, accusative, locative and instrumental cases; no forms are reported for 2nd and 3rd persons; no forms are reported for neuter gender; no forms are reported for other types of pronouns (e.g. possessive pronouns). For all reported forms, the Slovenska Bistrica and Ljubljana dialects exhibits only dual forms (rows 10-13/column II and III). The Nova Gorica dialect exhibits variable use of dual and plural forms in two of the cells and exclusive use of plural forms in the other two cells (rows 10-13/column IV). The Metlika dialect exhibits exclusive use of dual forms in one of the cells, variable use of dual and plural forms in one of the cells and exclusive use of plural forms in two of the cells (rows 10-13/column V).

Rows 1-9 report the use of dual/plural forms on nouns. Usage is reported for the masculine consonant-stem and *o*-stem 1st-declension nouns ‘son’ and ‘brother’ for nominative and dative (out of six cases for each), for the feminine *a*-stem and consonant-stem 1st-declension nouns ‘cow’ and ‘daughter’ for nominative and dative (out of six cases for each), and for the neuter *o*-stem 1st-declension noun ‘window’ for nominative (out of six cases). As seen from rows 1-9/column II, the Slovenska Bistrica dialect has only no plural-only cells and seven dual-only cells (for two cells, data is missing). As seen from rows 1-9/column III, out of the nine cells, the Ljubljana dialect has only dual forms in three cells, variably dual or plural forms in four cells, and only plural forms in two cells. As seen from rows 1-9/column IV, the Nova Gorica dialect has only dual forms in five cells, variably dual or plural forms in no cell, and only plural forms in four cells. As seen from rows 1-9/column V, the Metlika dialect has only dual forms in one cell, variably dual or plural forms in three cells, and only plural forms in four cells (for one cell, data is missing). Out of the four reported categories (verbs, pronouns, adjectives, and nouns), nouns are thus the category where the presence of the dual is most diverse inside each dialect and also most level across the four varieties.

As already mentioned, the difference between the four varieties is not such that the some would have special dual marking in all “semantically dual” grammatical contexts whereas others would always replace this with plural forms. Rather, the difference between the varieties is one of a scalar, greater/lesser presence of dual (see also Map S1). A simple summing up of the dual and plural forms is given in row 27 of Table S4. According to all three sums (sum of dual-only cells, of plural-only cells, and of dual/plural cells), the Slovenska Bistrica and Ljubjana varieties are found to use dual in more than half of the reported “semantically dual” contexts and the Nova Gorica and Metlika varieties use more plural in such contexts. Further, Slovenska Bistrica uses more dual and less plural forms in “semantically dual” contexts than the Ljubljana variety, while Nova Gorica uses more dual and less plural forms in “semantically dual” contexts than the Metlika variety.

In an attempt to quantify the presence of special dual marking in the three varieties, we summed up the cells with special dual marking for each variety. The dual/plural cells, which represent those “semantically dual” grammatical contexts for which variation between the dual and plural forms is reported, were counted as partially dual: in the actual sum, a plural-only cell was counted as 0, a dual-only cell as 1, and a dual/plural cell as 0.5. This yields the following result: The Slovenska Bistrica variety uses dual in 95.6% of the reported grammatical contexts, the Ljubljana variety uses dual forms in 73.1% of the reported grammatical contexts, the Nova Gorica variety uses dual forms in 36.5% of the reported grammatical contexts, and the Metlika variety uses dual forms in 16% of the reported grammatical contexts.

As is clear from the description of Table S4 above, its data represent a partial (forms are reported for a subset of grammatical contexts) and unweighted sample (relative frequency of individual grammatical contexts in child-directed speech). However, it is a random sample in the

sense that it was collected independently of our study and for purposes independent of our study, and it comes from the most comprehensive work on the use of the dual across Slovenian dialects. So in the absence of balanced and parallel corpora of dialectal Slovenian and of dialectal Slovenian child-directed speech, we resort to Jakop (2008) and our Jakop (2008)-based Table S4 and Map S1 to substantiate the claim that the Ljubljana and Slovenska Bistrica varieties of Slovenian uses considerably more dual than the varieties of Nova Gorica and Metlika, and that children from Ljubljana and Slovenska Bistrica dialect areas are exposed to more special dual marking than children from the Nova Gorica and Metlika dialect areas. This is confirmed independently by our results when testing children in the four locations on their knowledge of dual (see Results section in main text).

Socio-Economic Status Background

This section gives a short description of our four testing locations with respect to the socio-economic status of the relevant municipality. Ljubljana is an urban municipality in central Slovenia with a population of 282,000, Nova Gorica, and Metlika are urbanized municipalities on the western, and south-eastern fringes of Slovenia with populations of approximately 32,000 and 8,500, respectively, while Slovenska Bistrica is an urbanized municipality centrally located in the eastern part of Slovenia with a population of approximately 25,000. The reported figures were obtained from the Statistical Office of the Republic of Slovenia (www.stat.si) through its data portal (pxweb.stat.si). The municipalities are compared on the basis of two indicators: education and income.

Figure S1 below gives a comparison of the four testing locations in terms of education.

Figure S1: Level of education for residents of Ljubljana, Nova Gorica, Slovenska Bistrica, and Metlika (in 2014)

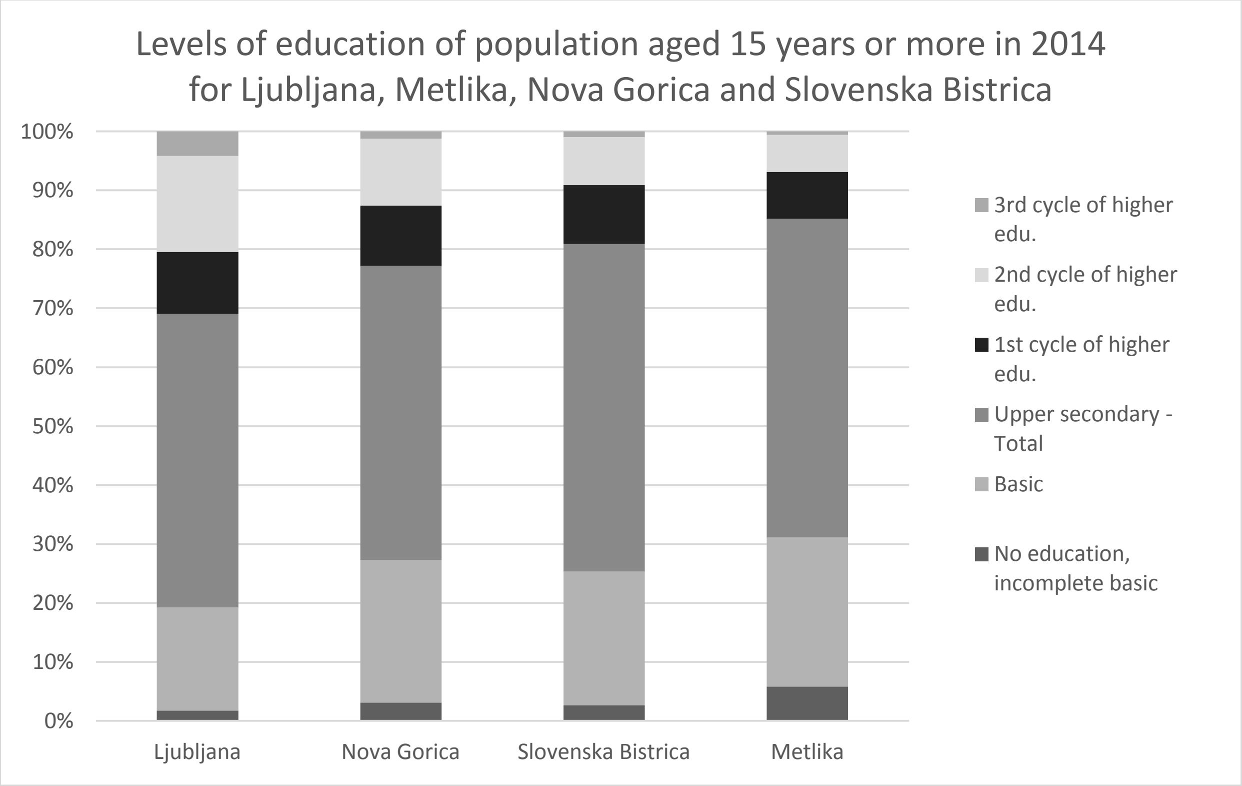

Figure S2 below shows average net monthly income in euros for each of the four testing locations in four consecutive years from 2010 to 2013.

Figure S2: Average net monthly income (euros) for residents of Ljubljana, Nova Gorica, Slovenska Bistrica, and Metlika. Horizontal line represents the average net monthly income for Slovenia.

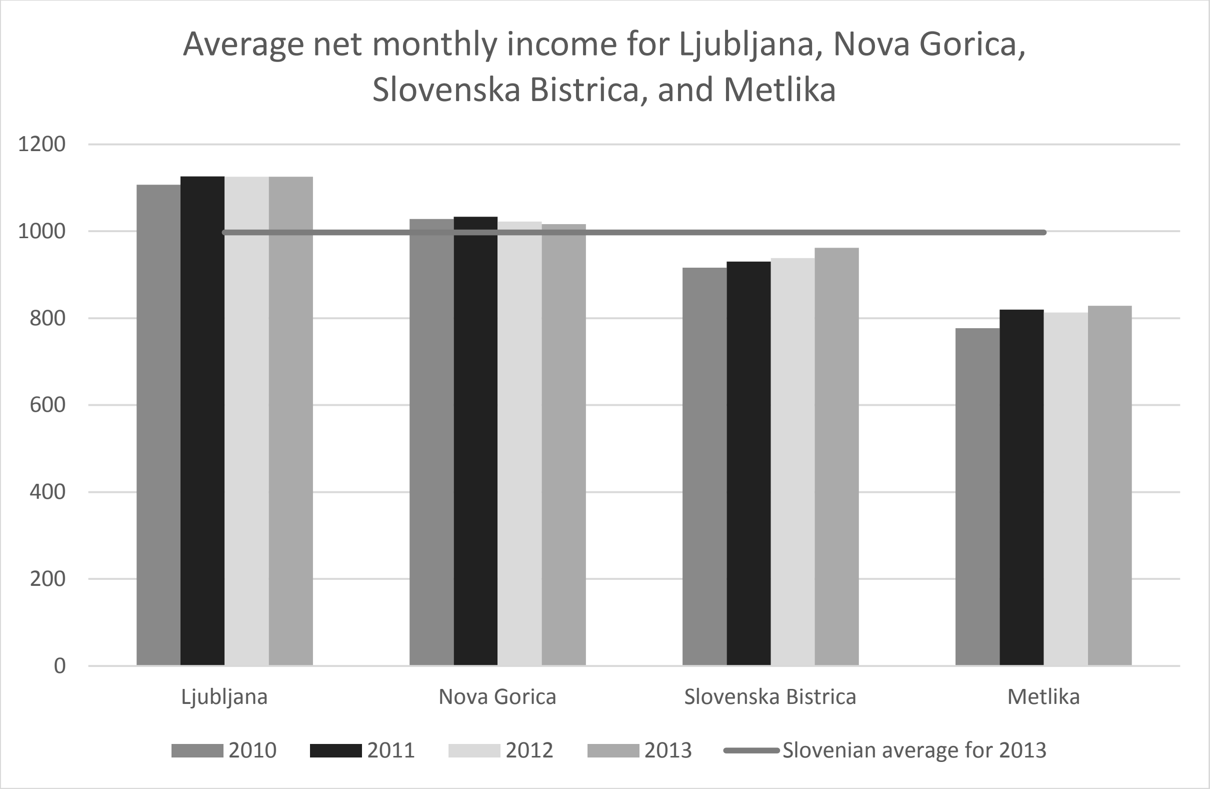

References:

1. Priestly, T. (1993). Slovene. In B. Comrie & G. G. Corbett (eds.) *The Slavonic Languages*. London: Routledge. pp. 388-451.
2. Corbett, G. G. (2000). *Number*. Cambridge: Cambridge University Press.
3. Derganc, A. (2003). The dual in Slovenian. In J. Orešnik & D. F. Reindl (eds.) *Slovenian from a typological perspective - Sprachtypologie und Universalienforschung* (Akademie Verlag, Berlin), Vol. 56.3. pp. 165-181.
4. Jakop, T. (2008). *Dvojina v slovenskih narečjih* [*The dual in Slovene dialects*]. Založba ZRC, ZRC SAZU: Ljubljana.
5. Herrity, P. (2000) *Slovene: A Comprehensive Grammar*. London, N.Y.: Routledge.
6. Marušič F. & Nevins A.I. (2010) Two Types of Neuter: Closest-Conjunct Agreement in the Presence of '5&Ups'. In W. Browne et al. (eds.) *Annual Workshop on Formal Approaches to Slavic Linguistics*, *The Second Cornell Meeting 2009*. Ann Arbor: Michigan Slavic Publications. pp. 301-317.
